# Flux control in the aging cascade

**DOI:** 10.18632/aging.202821

**Published:** 2021-03-13

**Authors:** Bernd Moosmann

**Affiliations:** 1 Evolutionary Biochemistry and Redox Medicine, Institute for Pathobiochemistry, University Medical Center of the Johannes Gutenberg University, Mainz, Germany

**Keywords:** free radicals, lipid peroxidation, longevity, radical chain reaction, rate-limiting step

Let us imagine that Gustav Embden (1874-1933), one of the ingenious discoverers of glycolysis, would have had modern transgenic techniques at hand and intended to use them to investigate the role of phosphoglycerate kinase (PGK) in the biochemical degradation of glucose to pyruvate. He would have probably overexpressed the enzyme 10x first, and he would not have seen any relevant change in the rate of pyruvate formation in the perfused working heart, whereas the addition of insulin would have shown a clear effect. He then would have generated 90% knockdown animals and again would not have seen any decrease in the rate of glycolysis. Hence, he would have confidently concluded that PGK was *not* involved in glycolysis. Thus, he would have arrived at an overtly wrong conclusion (merely hypothetical; sorry, Gustav!).

In essence, this is what we do today when we conclude from unsuccessful overexpression or knockdown studies of antioxidant enzymes [1,2] that free radicals were not involved in aging. We arrive at a wrong conclusion.

What is the mistake here, and what did Embden and his successors do better? First, they looked at the intrinsic chemical logic of the overall system. This should also be done in the study of aging. In particular, they recognized that steps can be involved and essential in a causal chain of (chemical) events even without ever being rate-limiting (or “flux-controlling”) for the overall passage through the chain of events. This principle applies to linear chains, branched chains, branching-converging chains and even cyclic chains. Because aging certainly represents an arrangement of causally chained elementary steps (of whatever type and complexity) [3], the decisive point will be to identify the flux-controlling steps of aging as narrowly as possible and then determine their control coefficients for the overall process.

Thus, the only thing we can learn from the fact that superoxide dismutase (SOD) modulation does not influence lifespan is that superoxide degradation is not flux-controlling for aging (in mice). This is still a valuable conclusion, even if it may not be particularly surprising: flux control is usually exerted by low-level, low-efficiency, or highly regulated enzymes, none of which applies to SOD. Moreover, if simple overexpression of SOD indeed would have had a measurable effect on lifespan, one might wonder why evolution has not yielded such a parsimonious solution before. Hence, it is quite unlikely that any isolated enzyme overexpression approach will ever substantially extend life in a species in which longevity is under positive selective pressure (like, arguably, in mammals). Extensive data support this generalization [2,4]. We have to grab for higher-hanging fruit.

Can we make any predictions about flux control in the aging cascade? Perhaps not in general, but clearly with respect to any potential radical involvement. All truly damaging radical reactions present themselves as chain reactions (in contrast to 1:1 stoichiometric reactions), since only chain reactions can destroy thousands of biomolecules following a single adverse event [5]. This conclusion also stands for non-radical chain reactions like the formation of AGEs. Chain reactions are generally characterized by three kinetically distinct steps: initiation, propagation, and termination (Figure 1). Notably, each of these steps can be independently flux-controlling for the velocity of the chain reaction as a whole [3]. Considering lipid peroxidation as case-in-point, the number of destroyed fatty acid molecules per time may be entirely governed by the rate of propagation and unrelated to the rate of initiation [5]. In fact, substantial evidence now points towards radical propagation as generally flux-controlling step not only of biological lipid peroxidation, but also of biological aging. Where does this idea come from?

**Figure 1:**
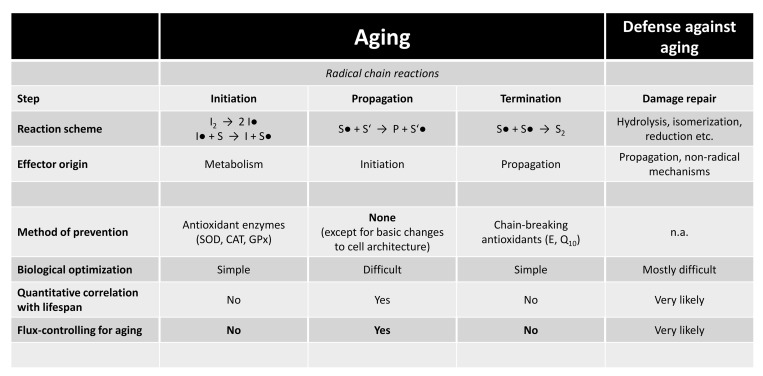
**Flux control in radical chain reactions in relation to biological aging.** Radical chain reactions have three kinetically independent steps: initiation, propagation and termination. The prevention of initiation is the designated task of antioxidant enzymes like SOD. Accelerating termination is the designated task of chain-breaking antioxidants like vitamin E. Propagation, though, has never been shown to be specifically influenced by any biomolecule [3]. It is only modulated by the availability of substrate (S) and the presence (or absence) of chain-transfer catalysts, which increase the propagation constant [3,8]. The two most robust evolutionary adaptations of long-lived animals, lowered polyunsaturated fatty acids [4], and fewer intramembrane cysteine residues [7], both slow down propagation, but have no direct connection to initiation or termination [3,8]. Damage repair is depicted for comparison, as one of several different defense strategies against aging. Very likely, damage repair also exerts flux control over aging (consider rapamycin), but the kinetically limiting steps are essentially unknown (e.g., recognition vs. excision vs. replacement). Abbreviations: I•, initiator radical; S•, substrate radical; P, product; SOD, superoxide dismutase; CAT, catalase; GPx, glutathione peroxidase; E, tocopherol; Q10, ubiquinone.

Inserting antioxidant enzymes like SOD into the general three-step model of radical chain reactions, one comes to realize that essentially all antioxidant enzymes only affect initiation, but not propagation or termination (Figure 1). Chain-breaking antioxidants like vitamin E or ubiquinone, in turn, exclusively affect termination, but not initiation or propagation. Incidentally, supplementation with antioxidants has been as unsuccessful in the modulation of lifespan as have antioxidant enzymes, indicating that neither radical initiation nor termination is flux-controlling for aging [3,4]. So, let us regard propagation. The daunting problem with propagation is that no discrete biological entity, be it an enzyme or a biochemical, has ever been shown to specifically affect it. Has evolution slept?

Not at all. Chemistry predicts at least three aspects to affect propagation: temperature, substrate concentration, and chain-transfer catalysis. Lowering temperature universally prolongs life in invertebrates but is a rather pleiotropic intervention. Surprisingly specific, though, are the “peroxidation index-lifespan correlation” [4,6], and the “cysteine-lifespan correlation” [7,8]. Long-lived animals exhibit lower contents of polyunsaturated fatty acids, which are the prototypic substrates of lipid peroxidation [4,5]. In perfect complementation, long-lived animals also exhibit lower numbers of intramembrane cysteine residues (in mitochondria), which avoids chain-transfer catalysis by thiol groups that would otherwise accelerate radical propagation between fatty acids [8]. Both correlations are valid across phylogenetic boundaries and are by far more robust than any runner-up. Moreover, basic experimental evidence of causality has been obtained for each of them [6,8]. Since both adaptations alter the complex molecular architecture of the cell, it becomes increasingly intelligible why longevity is such a slowly evolving trait. As Gustav Embden probably would have predicted correctly, flux control in the aging cascade is exerted by the step that is the most difficult one to optimize, by nature or by man: propagation.

## References

[1] Pérez VI, et al. Aging Cell. 2009; 8:73–75. 10.1111/j.1474-9726.2008.00449.x 19077044 PMC2667893

[2] Pérez VI, et al. Biochim Biophys Acta. 2009; 1790:1005–14. 10.1016/j.bbagen.2009.06.003 19524016 PMC2789432

[3] Kunath S, Moosmann B. Geroscience. 2020; 42:857–66. 10.1007/s11357-019-00058-2 30809734 PMC7287003

[4] Hulbert AJ, et al. Physiol Rev. 2007; 87:1175–213. 10.1152/physrev.00047.2006 17928583

[5] Zimniak P. Free Radic Biol Med. 2011; 51:1087–105. 10.1016/j.freeradbiomed.2011.05.039 21708248 PMC3156362

[6] Shmookler Reis RJ, et al. Aging (Albany NY). 2011; 3:125–47. 10.18632/aging.100275 21386131 PMC3082008

[7] Moosmann B, Behl C. Aging Cell. 2008; 7:32–46. 10.1111/j.1474-9726.2007.00349.x 18028257

[8] Kunath S, et al. Redox Biol. 2020; 36:101628. 10.1016/j.redox.2020.101628 32863215 PMC7365990

